# Predictors for Pulmonary Tuberculosis Outcome and Adverse Events in an Italian Referral Hospital: A Nine-Year Retrospective Study (2013–2021)

**DOI:** 10.5334/aogh.3677

**Published:** 2022-04-26

**Authors:** Francesco Di Gennaro, Rossana Lattanzio, Giacomo Guido, Aurelia Ricciardi, Roberta Novara, Giulia Patti, Sergio Cotugno, Elda De Vita, Gaetano Brindicci, Michele Fabiano Mariano, Luigi Ronga, Carmen Rita Santoro, Federica Romanelli, Stefania Stolfa, Roberta Papagni, Davide Fiore Bavaro, Giusi De Iaco, Annalisa Saracino

**Affiliations:** 1 Clinic of Infectious Diseases, University of Bari “Aldo Moro”, Department of Biomedical Sciences and Human Oncology, Bari, IT; 2 Microbiology and Virology Unit, University of Bari, University Hospital Policlinico, Bari, IT

## Abstract

**Background:**

The COVID-19 pandemic has undone years of progress in providing essential TB services and controlling the TB burden. Italy, a low TB burden country, has an incidence of 7.1 cases per 100,000 people. To control the TB spreading in Italy is critical to investigate the characteristics of patients with the worst outcomes and the highest risk of adverse events related to antituberculosis therapy. Therefore, we conducted a large retrospective study in TB patients admitted to the Clinic of Infectious Diseases University of Bari, Italy, in order to describe the clinical presentation and the factors associated with adverse events and outcomes.

**Methods:**

We retrospectively evaluated the patients admitted to the Clinic of Infectious Diseases from January 2013 to 15 December 2021. We stratified our cohort into two groups: <65 years of age and ≥65 years in order to assess any differences between the two groups. Two logistic regression models were implemented considering the dependent variables as: (I) the adverse events; and (II) the unsuccessful treatments.

**Results:**

In total, 206 consecutive patients [60% (n = 124) M, median age 39 years, range 16–92] were diagnosed and admitted with TB at Clinic of Infectious Diseases. Of the whole sample, 151 (74%) were <65 years and 55 (26%) were ≥65. Statistically significant differences between the two groups were detected (*p-value* < 0.05) for nationality (*p-value =* 0.01), previous contact with TB patient (*p-value* = 0.00), type of TB (*p-value* = 0.00), unsuccessful treatment (*p-value* = 0.00), length of hospitalization (*p-value* = 0.02) and diagnostic delay (p-value = 0.01). Adverse events related to TB drug regimen were reported in 24% (*n* = 49). Age < 65 years (O.R. = 3.91; 95% CI 1.72–4.21), non-Italian nationality (O.R. = 4.45; 95% CI 2.22–4.98.), homeless (O.R. = 3.23; 95% CI 2.58–4.54), presence of respiratory symptoms (O.R. = 1.23; 95% CI 1.10–1.90), diagnostic delay (O.R = 2.55; 95% CI 1.98–3.77) resulted associated with unsuccessful treatment outcome (death, failure or lost to follow up). Finally, age < 65 years (O.R. = 1.73; 95% CI 1.31–2.49), presence of pulmonary TB (O.R. = 1.15; 95% CI 1.02–1.35), length of hospitalization (O.R. = 1.82; 95% CI 1.35–2.57) and TB culture positive (O.R. = 1.35; 95% CI 1.12–1.82) were associated with adverse events in our populations.

**Conclusions:**

The pharmacological approach alone seems insufficient to treat and cure a disease whose ethiopathogenesis is not only due to the *Mycobacterium tuberculosis*, but also to the poverty or the social fragility. Our data suggest that young foreigners, the homeless, and the people with low social and economic status are at higher risk of an unfavorable outcome in low incidence TB countries. Targeted actions to support this highly vulnerable population both in terms of outcome and occurrence of adverse events are needed.

## Background

Tuberculosis (TB) is still one of the world’s leading killers and one of the most serious public health issues, with 10 million new cases diagnosed each year and 1.5 million deaths [1]. Thirty high TB burden countries, the majority of which are in Sub-Saharan Africa, account for nearly 90% of all TB cases each year [2]. Unfortunately, the COVID-19 pandemic has undone years of progress in providing essential TB services and controlling the TB burden [3,4]. In fact, a significant drop in new TB diagnoses, down 18% from 7.1 million in 2019 to 5.8 million in 2020, was observed, bringing this score back to around ten years ago [1,2]. Furthermore, there has also been a 15% decrease in access to TB care, an increase in diagnostic delay and worse outcomes. These indicators are also expected to deteriorate further until 2022, effectively undoing the previous decade’s significant efforts and commitments to combat TB [1,2]. In most of the 30 high TB burden countries, TB notification rate is around 150–400 cases per 100 000 population [4]. In low endemic countries such as Italy, TB mainly affects vulnerable populations such as immigrants, homeless, gypsies, prisoners and elderly (≥ 65 years old) [5]. Italy, a low TB burden country, has an incidence of 7.1 cases per 100 000 people. Moreover, approximately 60% of cases are due to the migrant population coming from areas of high TB incidence (such as Africa, Asia and Eastern Europe). The most foreigners come from African countries (Senegal, Ghana, Nigeria, Cameroon), Eastern Europe (Albania, Bulgaria, Ukraine, and Poland), and the Indian subcontinent (India, Bangladesh, and Pakistan), where the epidemiological situation is complicated by the spread of multi drug resistant (MDR) or extensively drug-resistant (XDR) strains [6,7]. Furthermore, the impact of the COVID-19 pandemic on TB services is estimated as dramatic, particularly in countries where TB-related healthcare personnel have been allocated to the COVID-19 emergency. The WHO warned that the COVID-19 pandemic’s interruption of TB services might result in fewer TB diagnosis, longer diagnostic delays, and increase of the TB mortality and the onset TB MDR [1]. For this reason, it is critical to investigate the characteristics of patients with the worst outcomes and the highest risk of adverse events related to antituberculosis therapy with the aim to effectively control TB spreading in Italy.

Therefore, we conducted a large retrospective study by evaluating patients with pulmonary TB admitted to the University Clinic of Infectious Diseases, University of Bari, Apulia region, south of Italy in order to describe the clinical presentation and the factors associated with adverse events and outcomes.

## Materials and Methods

### Study Design and Patients

We performed a retrospective study in patients diagnosed with pulmonary TB admitted to the University Clinic of Infectious Diseases University of Bari, Bari, Italy, from 1 January 2013 to 15 December 2021. We retrospectively evaluated data from each patient admitted in our hospital with pulmonary active TB. According to the WHO guidelines, enrolled patients were classified as “active pulmonary TB” if the diagnosis was based: (1) on a positive culture for *M. tuberculosis* from a respiratory sample (sputum or bronchoalveolar lavage) or other biological specimens; (2) on positive *M. tuberculosis* nucleic amplification test NAT (Xpert^®^ MTB/RIF, Cepheid, USA) from biological specimens (without culture confirmation); or (3) on histopathological findings consistent with TB and presence of acid fast bacilli (AFB) in a tissue sample. Moreover, patients were classified as “clinical TB” if the diagnosis was based on clinical and radiologic criteria (having excluded other diseases) including appropriate response to standard anti-TB therapy.

Patients were treated according to the institutional protocol drawn up following the WHO TB guidelines [8]. The first week of treatment was spent in the hospital until AFB sputum conversion was accomplished on three consecutive negative samples taken over the course of a week. During their stay in the hospital, patients received direct observed therapy. Patients were subsequently followed monthly on ambulatory care by certified TB experts after discharge for the duration of their treatment. Until the treatment was finished, laboratory tests were performed almost weekly, or as needed depending on the clinical status. Medical advice was provided about the likelihood of adverse drug events and the need of reporting any adverse event to their doctor as soon as possible. Patients were also provided a booklet with information on the most prevalent anti-TB drugs adverse effects.

### Data Collection

Patient demographics; admission and dis-charge/death dates; and clinical variables: symptoms, tuberculosis diagnosis, M. tuberculosis drug resistance, TB location, treatment regimen, adverse events (type, severity, management), and outcomes were the key data sources. The severity of adverse events was defined as light (asymptomatic laboratory findings only; modest signs/symptoms; no medical intervention necessary), moderate (necessitating limited non-invasive intervention), and severe (significant symptoms requiring hospitalization) [9].

### Statistical Analysis

No formal sample size was calculated *a priori*, since the study included all patients admitted during the study period. Continuous data were expressed as median and interquartile range (IQR), and categorical data as numbers and percentages. Chi-squared test or Fisher’s exact test as appropriate were used to compare categorical variables. We stratified our cohort into two groups: <65 years of age and ≥65 years in order to evaluate any differences between these two groups.

Two logistic regression models were implemented considering the dependent variables as: (1) the adverse events; and (2) unsuccessfully treated (died, lost to follow up and failed patients) while each of the available factors were considered as independent variables (univariate analysis). All the factors with a *p-value* < 0.10 at the univariate analyses were included in the models. Multicollinearity among covariates was assessed through the variance inflation factor (VIF), taking a value of two for excluding a covariate. However, no variable was excluded according to the previous criterion.

Odds ratios (ORs) as adjusted odds ratios (Adj–ORs) with their 95% confidence intervals (CIs) were used to measure the association between factors at the baseline (exposure) and treatment failure (outcome).

All two-tailed test *p value* less than 0.05 were considered statistically significant. Statistical analysis was performed using STATA V.13.

## Results

Between January, 1 2013 and December, 15 2021, 206 consecutive patients [60% (n = 124) M, median age 39 years, range 16–92] were diagnosed and admitted with TB at the Clinic of Infectious Diseases, and included in the study. Of the whole sample, 151 (74%) were <65 years. Median age was 32 years (16–63) and 71 years (65–92) for <65 years group and ≥65 years one, respectively. Among them, 51% (n = 105) had non-Italian nationality, 8% (n = 17) were homeless, and 65% (n = 135) had a pulmonary localization of TB. Notably, 60% (n = 124) had respiratory symptoms. Furthermore, 68% (n = 140) were culture positive and 16% (n = 34) showed at least one drug resistance. Fifty-seven percent (n = 117) successfully completed the treatment, while 1% (n = 4) died due to a TB-related cause. At the time of the study, treatment was still ongoing 10% (n = 20) of the sample. Furthermore, the treatment success rate in ≥65 years group was 84% (n = 46), while in <65 years was 45% (n = 71).

***Table 1*** shows the characteristics of all participants on the whole sample and stratified by age-classes <65 or ≥65 years old. Differences in distribution of the variables collected between <65 or ≥65-year-old groups emerged (*p-value* < 0.05) for nationality (*p-value* = 0.01), previous contact with TB patient (*p-value* = 0.00), type of TB (*p-value* = 0.00), unsuccessful treatment (*p-value* = 0.00), length of hospitalization (*p-value* = 0.02) and diagnostic delay (*p-value* = 0.01) as shown in ***Table 1***.

**Table 1:** Characteristics of participants stratified by age-classes <65 or ≥65 years old.


		ADMITTED PATIENTS			*P*-VALUE
	
		TOTAL *N. 206 (100%)*	AGED < 65 *N. 151 (100%)*	AGED ≥ 65 *N. 55 (100%)*	

Sex	M	124 (60)	91 (74)	33 (26)	0.9

	F	82 (40)	60 (75)	22 (25)	

	Age Median (IQR)	39 (16–92)	32 (16–63)	71 (65–92)	0.00

Nationality	Italian	101 (49)	54 (53)	47 (47)	0.01

	Non-Italian	105 (51)	97 (94)	8 (6)	

	Homeless	17 (8)	17 (100)	0 (0)	NA

	HIV + status	6 (3)	5 (84)	1 (16)	0.9

	Previous contact with tb patient	60 (30)	52 (88)	8 (12)	0.00

	Hospital stay, median	44,5	43 (7–230)	24 (4–45)	0.02

	Diagnostic delay, median	76	127 (2–450)	43 (5–120)	0.01

Type	Pulmonary TB	135 (65)	107 (80)	28 (20)	0.00

	Extrapulmonary TB	64 (31)	38 (60)	26 (40)	0.01

	Miliary TB	7 (4)	6 (86)	1 (14)	0.7

	Respiratory symptoms	124 (60)	101 (81)	25 (19)	0.02

Type of diagnosis	Culture positive	140 (68)	107 (77)	33 (23)	0.03

	Radiological	42 (20)	31 (74)	11 (26)	0.03

	NAT	5 (2)	2 (40)	3 (60)	0.2

	Histological	19 (9)	10 (48)	9 (52)	1

	IGRA test	123 (62)	97 (78)	26 (22)	0.08

Initial Therapeutic Scheme, n (%)	R+H+E+Z	166 (80)	123 (75)	44 (25)	0.9

	Drug regimen without Z Including Amikacin	5 (2)	2 (40)	3 (60)	0.6

	Drug without Z regimen including fluoroquinolone	1 (1)	1(100)	0 (0)	NA

Resistance Pattern	Monoresistance	34 (16)	25 (73)	9 (27)	0.3

	H	18 (9)	14 (77)	4 (23)	0.7

	R	13 (6)	7 (54)	5 (46)	0.5

	Z	3 (1)	2 (66)	1 (34)	1

	MDR	9 (4)	9 (100)	0 (0)	0.1

Adverse events and management, n (%)	Adverse events	49 (25)	36 (73)	13 (27)	0.3

	Therapeutic Shift	24 (12)	19 (80)	6 (20)	0.5

Outcomes	Exitus	4 (1)	3 (75)	1 (25)	0.8

	Successful treatment	117 (57)	71 (60)	46 (40)	0.1

	Unsuccessful treatment	65 (32)	61 (94)	4 (6)	0.00

	Treatment on going	20 (10)	16 (66)	4 (34)	0.1


Adverse events related to the TB drug regimen were reported in 24% (n = 49) of patients. Out of the 49 patients who reported an adverse event, 55% (n = 27) showed a liver disease. In 51% (n = 25) of the 49 patients, the event required minimal non-invasive intervention classified as mild while 49% (n = 24) of cases required a suspension of the suspect drug and change of the treatment. Further characteristics of adverse events reported in the sample are shown in ***Table 2***.

**Table 2:** Characteristics of adverse events in the 49 patients who reported them.


	CHARACTERISTICS	TOTAL N. 49 (100%)

**Type of Adverse events, n (%)**	Hepatitis	27 (55)

	Neurological	5 (11)

	Ocular damage/decrease in visual acuity	5 (11)

	Itching/skin rash	11 (22)

	Acute renal failure	1 (1)

**Severity of Adverse events, n (%)**	Mild	25 (51)

	Moderate	18 (37)

	Severe	6 (22)

**Adverse events management, n (%)**	Therapeutic shift	24 (49)

	Temporary suspension of all treatment	9 (18)

	Support therapy and no change of treatment	16 (33)


The multivariate logistic model on unsuccessful outcome considered the effects of age, gender, nationality, homelessness, presence of respiratory symptoms, diagnostic delay, length of hospitalization, acid-fast bacilli smear positive, drug resistance, treatment, TB localization (lung or extrapulmonary) and TB culture positivity. Significant predictors of unsuccessful outcome are reported in ***Table 3***. Age < 65 years (O.R. = 3.91; 95% CI 1.72–4.21), non-Italian nationality (O.R. = 4.45; 95% CI 2.22–4.98.), homelessness (O.R. = 3.23; 95% CI 2.58–4.54), presence of respiratory symptoms (O.R. = 1.23; 95% CI 1.10–1.90), diagnostic delay (O.R = 2.55; 95% CI 1.98–3.77) resulted associated with unsuccessful treatment outcome (death, failure or lost to follow up), as reported in ***Table 3***.

**Table 3:** Predictors of unsuccessful treatment for active pulmonary tuberculosis.


CHARACTERISTICS	UNIVARIATE ANALYSIS O.R.	MULTIVARIATE ANALYSIS ADJ-O.R.

Age < 65	1.22 (0.98–1.64)	3.91 (1.72–4.21)*

Female	0.48 (0.16–0.90)	0.68 (0.16–1.10)

Non-Italian nationality	1.40 (1.28–1.76)	4.45 (2.22–4.98)*

Homeless	1.51 (1.28–2.03)	3.23 (2.58–4.54)*

Diagnostic delay	1.72 (1.08–2.01)	2.55 (1.98–3.77)*

Length of hospitalisation	1.15 (0.68–1.54)	1.42 (0.85–1.87)

Pulmonary TB	1.21 (0.28–1.23)	1.13 (0.88–1.94)

Extrapulmonary TB	1.14 (0.88–1.58)	1.84 (0.91–2.78)

Respiratory symptoms	1.26 (0.85–1. 72)	1.23 (1.10–1.90)*

Culture positive	1.64 (0.38–1.78)	1.24 (0.38–1.48)

Monoresistance, n	0.35 (0.12–0.60)	0.75 (0.45–1.34)

MDR	1.21 (0.89–1.73)	–

R + H + E + Z	0.79 (0.68–1.21)	1.10 (0.83–2.21)

Drug regimen without Z including Amikacin	0.59 (0.48–1.21)	–


**Legend** R: Rifampicin H: Isoniazid E: Ethambutol Z: Piraldine TB: Tuberculosis.

The multivariate logistic model on the adverse events considered the effects of age, gender, nationality, homelessness, presence of respiratory symptoms, diagnostic delay and length of hospitalization, acid-fast bacilli smear positive, drug resistance, treatment and TB culture positivity, TB localization (lung or extrapulmonary). Age < 65 years (O.R. = 1.73; 95% CI 1.31–2.49), presence of pulmonary TB (O.R. = 1.15; 95% CI 1.02–1.35), length of hospitalization (O.R. = 1.82; 95% CI 1.35–2.57) and TB culture positive (O.R. = 1.35; 95% CI 1.12–1.82) resulted associated with adverse events in our populations, as reported in ***Table 4***.

**Table 4:** Predictors of adverse events for active pulmonary tuberculosis.


CHARACTERISTICS	UNIVARIATE ANALYSIS O.R.	MULTIVARIATE ANALYSIS ADJ-O.R.

Age <65	1.18 (0.81–3.47)	1.73 (1.31–2.49)*

Female	0.48 (0.26–1.09)	0.38 (0.16–1.05)

Non-Italian nationality	1.40 (0.88–1.76)	0.95 (0.82–1.59)

Homeless	0.75 (0.55–1.06)	0.88 (0.55–1.18)

HIV status	0.44 (0.28–1.06)	–

Diagnostic delay	1.10 (0.68–1.41)	1.45 (0.75–1.97)

Length of hospitalisation	1.25 (0.88–1.71)	1.82 (1.35–2.57)*

Pulmonary TB	0.76 (0.38–1.08)	1.15 (1.02–1.35)*

Extrapulmonary TB	0.70 (0.48–1.16)	0.83 (0.62–1.16)

Respiratory symptoms	1.21 (0.88–1.76)	0.95 (0.82–1.58)

Culture positive	1.20 (0.78–1.56)	1.35 (1.12–1.82)*

Monoresistance, n	0.64 (0.48–0.96)	0.95 (0.82–1.29)

MDR	1.40 (0.88–1.76)	–

R + H + E + Z	1.04 (0.88–1.46)	0.85 (0.72–1.64)

Drug regimen without Z including Amikacin	0.39 (0.28–0.56)	–


**Legend** R: Rifampicin H: Isoniazid E: Ethambutol Z: Piraldine TB: Tuberculosis.

**Figure 1:**
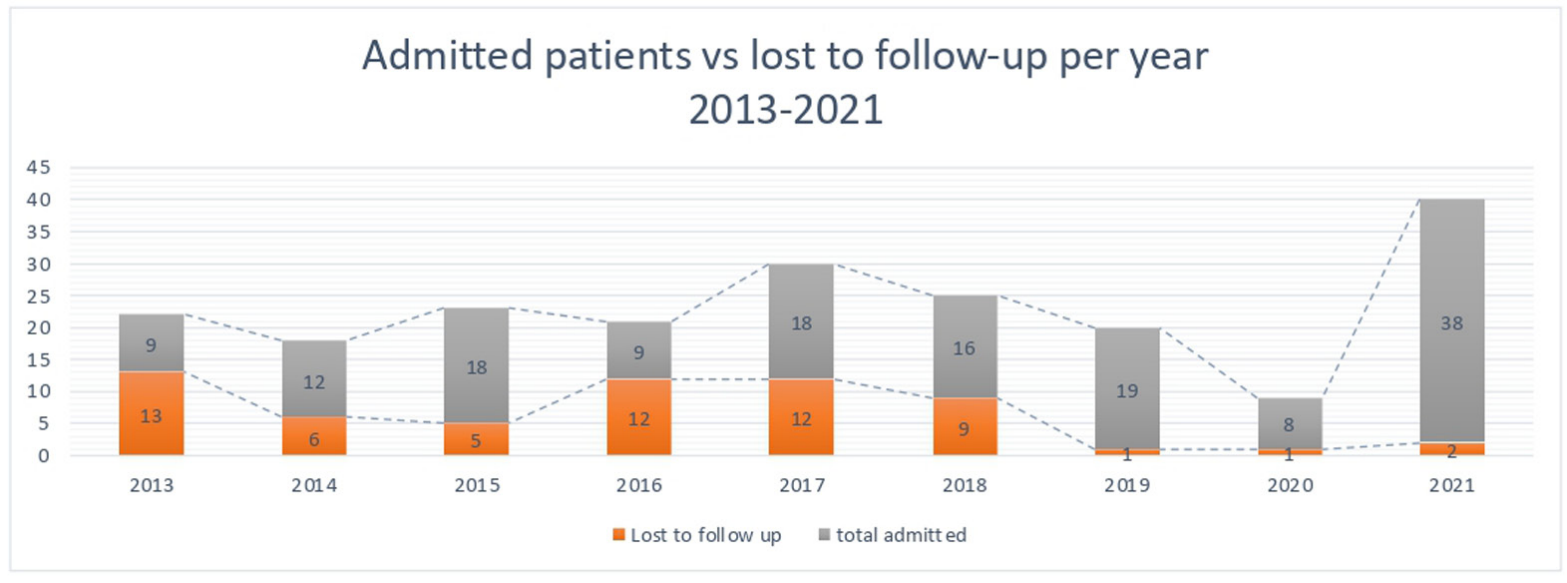
2013–2021 trend of admitted patients and lost to follow up.

Furthermore, ***Figure 1*** shows the temporal trend (2013–2021) of admitted TB patients and lost one to follow-up.

## Discussion

Our study describes a cohort of patients admitted in a referral hospital for TB in Italy referred to a large time period (2013–2021). According to the WHO definitions, 140 out of 206 patients were microbiologically confirmed with pulmonary TB. A global successful treatment was reported in half of our sample, while one out four reported an adverse event related to TB drug regimen and 16% presented at least of one drug resistance. Stratifying our data by age classes <65 or ≥65 years old, differences were found in: nationality, previous contact with TB, length of hospitalization, diagnostic delay.

Quite surprising are the results concerning the factors associated with an unsuccessful outcome. In fact, in our sample, age < 65 years, non-Italian nationality, homelessness, presence of respiratory symptoms, and a diagnostic delay resulted associated with an unsuccessful treatment outcome (death, failure or lost to follow up).

In line with European data, in our cohort there is a slight prevalence of male subjects [10,11] and almost three-quarters of our cohort belonged to the younger group with a median age of 39 years; our study population is younger than would be expected in low TB burden countries in which the geriatric population represents a large reservoir of TB infection [12,13].

This result is due to the fact that 51% of our patients were foreigners and 94% of them were less than 65 years. In our case, the role of the migrant population is greater than in the rest of Europe. In fact, the latest 2021 report records that approximately one third (34.5%) of TB cases reported in the EU/EEA in 2019 were of foreign origin [10,14].

Several authors showed as being elderly is a risk factor for a worse outcome and expose to a major risk of adverse events [15,16]. This contradiction with our data may be explained on the one hand by the good therapeutic and follow-up management towards this risk category and on the other hand may have been influenced by the fact that highly vulnerable populations such as migrants, homeless and foreigners were under 65 years of age [13,17]. In fact, as showed by several experiences, foreigners, homelessness and more generally a low socio-economic level impact in worsening outcomes [18]. In fact, a recent meta-analysis of more than 400 000 patients shows that the risk of MDR is at least twice as high in populations with a lower socio-economic level [19].

In EU countries, the average treatment success rate for active TB has been reported as 60%–87%. Our data globally showed a 57% of successful treatment, but with a notably difference between the two groups [10]. In fact, success treatment rate in ≥65-year-old group was 84% (n = 46), while in <65-year-olds it was 45% (n = 71). These underlines how we are fully above the European average for the elderly TB population while our data represent below average for the <65 years group representing the great vulnerability and fragility of this population for the greater presence in this group of migrants, homeless people with known social fragilities which are then reflected in health outcomes [20,21].

Moreover, Apulia and the other regions of South Italy are located in the middle of the Mediterranean migration route with high prevalence of migrant population that represents a pivotal key to TB burden control [22].

Furthermore, in our cohort one out four patients showed an adverse event related to TB drug regimen. Around half of them showed a hepatitis and half of these patients changed treatment. Factors associated with adverse events were age < 65 years, pulmonary TBC, length of hospitalization and TB culture positive.

This is in contradiction with the literature which shows a variable percentage of 15–75% of adverse events in the over 65 and especially over 75 population [23,24]. This could be explained by careful therapeutic and clinical management for the elderly, in a multidisciplinary approach with specialists for the other comorbidities of these patients, and in a close up clinical and hematochemical followed up [25]. Regardless drugs, pyrazinamide was responsible for the majority of adverse reactions as showed in literature, but unfortunately we do not have data on drugs due to toxicity [26,27].

Furthermore, our data show that patients under 65 have a higher diagnostic delay than patients over 65 (76 days vs. 43 days) and also higher hospital length of stay (43 vs. 24). Our study found a median TD of 76 days in line with other regions of our country (77.5 days in Lazio, greater instead than Emilia Romagna with a TD of 65 days) [28]. This finding is also in agreement with the TD estimated in a review conducted on 58 studies, performed in both low- and high-endemic countries, which reported a delay within the range of 60 to 90 days [29].

There may be several reasons for what has been observed: in a country with low endemicity for tuberculosis there is a low awareness of the disease both by the patients and by the general practitioners; in our case series, the extra-pulmonary forms, which are characterized by a more specific symptomatology and do not present the classic clinical pattern of the respiratory forms, are more frequent in the young population. Moreover, a large part of the young population is represented by migrants who, despite the presence in our country of a free national health system, may experience difficulties due to the condition of irregular migrants without access to the general practitioners and because of this the diagnosis is often only made after the access to the public hospitals due to the manifestations of the advanced clinical forms.

Another statistically significant difference between the young and elderly population was found in the length of stay. In the younger the average length of stay is 43 days (with a wide range from 7 to 230 days) versus 24 days in the older population (from 5 to 120 days). As already observed in other studies, the risk of hospitalization is not only influenced by clinical factors, but also by social determinants and high hospitalization times is generally greater in: migrants who have been in the country of arrival for more than two years, the homeless and patients with multidrug-resistant TB [30,31]. However, some studies conducted in countries with low TB incidence reported that more than three quarters of patients are initially treated as inpatients and in many cases this is an unreasonable choice with longer hospitalization times than strictly necessary. This result is in line with the study of Aslam et al., which estimated that about 38% of hospitalizations were avoidable [32].

Furthermore, ***Figure 1*** also allows for two reflections. The first is the reduction in TB diagnoses during the SARS CoV2 pandemic, as well as the lockdown periods with interruption and disruption of TB services due to the pandemic’s need and workload. As several authors have reported, this may have a negative impact on Tb burden control, with an increase in clinical severity, diagnostic delay, and, more broadly, a reduction in TB control, pushing the results obtained in the fight against tuberculosis back ten years.

The second reflection is on the potential impact of establishing a tuberculosis-specific medical and nursing team for those who were lost to follow-up. In fact, as of 2019, a team specializing in tuberculosis is caring for patients in our hospital, which could explain the significant decrease in lost to follow-up over the last three years. Of course, studies focusing on this potential impact are required to demonstrate the intervention’s potential effectiveness.

We acknowledge that our study has some limitations. First, the inclusion of the patients diagnosed at a single institution may limit the generalizability of the results. Moreover, due to the study’s retrospective nature, other factors potentially influencing outcomes, such as lack of comorbidity data, radiological findings, and other risk factors, were not considered. Furthermore, in our study, we only considered total diagnostic delay, with no distinction made between healthcare system and patient delays.

## Conclusion

In conclusion, our data suggest that young foreigners, the homeless, and people with low social and economic status are at higher risk of an unfavorable outcome in low incidence TB countries. According to the WHO, one of the pilot keys for high-income countries to control TB burden is to target TB vulnerable groups [33,34,35,36]. Targeted actions to support this highly vulnerable population both in terms of outcome and occurrence of adverse events are needed. The pharmacological approach alone seems insufficient to treat and cure a disease whose ethiopathogenesis is not only related to *the Mycobacterium Tuberculosis*, but also to the poverty. The establishment of a dedicated medical and nursing team for tuberculosis could improve outcomes and reduce lost to follow-up for this vulnerable population, but more integrated social actions are required. Hospitalization in tertiary referral hospitals with clinical multidisciplinary expertise in TB diagnosis and treatment can be useful to improve outcome in the elderly. Concentrating elderly TB patients to referral facilities is important to ensure a better outcome for this fragile population, which is different from the previous one and requires dedicated expertise on TB care.

Furthermore, new treatments and short regimens should be specifically evaluated in vulnerable populations to increase adherence, reduce the risk to lost to follow up, reduce the pill burden and treatment time, and improve outcome and safety. Future research is therefore needed to evaluate targeted interventions to assist vulnerable populations and to effectively control and eliminate tuberculosis as a goal of sustainable development.

## Data Accessibility Statement

All authors confirm that the data supporting the findings of this study are available within the article. All datasets generated and/or analyzed during the current study are available from the corresponding author on request. Data request can be made through the corresponding author’s email.

## Abbreviations

TB: Tuberculosis

WHO: World Health Organization

EU: European Countries

MDR: multi drug resistant

XDR: extensively drug-resistant

R: Rifampicin

H: Isoniazid

E: Ethambutol

Z: Piraldine

M.t: Mycobacterium tuberculosis

PD: patient delay

HD: hospital delay

DD: diagnostic delay

TD: total delay

## References

[1] World Health Organization (WHO). Global Tuberculosis Report 2021. Geneva: WHO; 2021. https://www.who.int/publications/i/item/9789240037021

[2] Chakaya J, Khan M, Ntoumi F, et al. Global Tuberculosis Report 2020 – Reflections on the Global TB burden, treatment and prevention efforts. Int J Infect Dis. 2021 Mar 11; S1201–9712(21); 00193–4. DOI: 10.1016/j.ijid.2021.02.107 PMC8433257 33716195

[3] Togun T, Kampmann B, Stoker NG, Lipman M. Anticipating the impact of the COVID-19 pandemic on TB patients and TB control programmes. Ann Clin Microbiol Antimicrob. 2020 May 23; 19(1): 21. DOI: 10.1186/s12941-020-00363-1 32446305 PMC7245173

[4] Di Gennaro F, Gualano G, Timelli L, et al. Increase in Tuberculosis Diagnostic Delay during First Wave of the COVID-19 Pandemic: Data from an Italian Infectious Disease Referral Hospital. Antibiotics (Basel). 2021 Mar 8; 10(3): 272. DOI: 10.3390/antibiotics10030272 33800406 PMC7998965

[5] Pontarelli A, Marchese V, Scolari C, et al. Screening for active and latent tuberculosis among asylum seekers in Italy: A retrospective cohort analysis. Travel Med Infect Dis. 2019 Jan–Feb; 27: 39–45. DOI: 10.1016/j.tmaid.2018.10.015 30347248

[6] Villa S, Codecasa LR, Faccini M, et al. Tuberculosis among asylum seekers in Milan, Italy: epidemiological analysis and evaluation of interventions. Eur Respir J. 2019 Oct 31; 54(4): 1900896. DOI: 10.1183/13993003.00896-2019 31413161

[7] Boudville DA, Joshi R, Rijkers GT. Migration and tuberculosis in Europe. J Clin Tuberc Other Mycobact Dis. 2020 Jan 7; 18: 100143. DOI: 10.1016/j.jctube.2020.100143 31956700 PMC6957817

[8] World Health Organization (WHO). Consolidated guidelines on tuberculosis. Geneva: WHO; 7 July 2021. https://www.who.int/publications/i/item/9789240029415

[9] Palmieri F. Per il Gruppo di Lavoro Tubercolosi-INMI “L. Spallanzani” Percorso Diagnostico Terapeutico Assistenziale Sulla Gestione del Paziente con Infezione/Malattia Tubercolare. Revisione N. 8/Gennaio 2020. Accessed 30 November 2021. http://www.inmi.it/servizio/protocolli_e_linee_guida.

[10] European Centre for Disease Prevention and Control & World Health Organization. Regional Office for Europe. (2020). Tuberculosis surveillance and monitoring in Europe 2020: 2018 data. World Health Organization. Regional Office for Europe. https://apps.who.int/iris/handle/10665/331530.

[11] Kraef C, Bentzon A, Panteleev A, et al. Delayed diagnosis of tuberculosis in persons living with HIV in Eastern Europe: associated factors and effect on mortality-a multicentre prospective cohort study. BMC Infect Dis. 2021 Oct 6; 21(1): 1038. DOI: 10.1186/s12879-021-06745-w 34615474 PMC8496077

[12] Li J, Chung PH, Leung CLK, Nishikiori N, Chan EYY, Yeoh EK. The strategic framework of tuberculosis control and prevention in the elderly: a scoping review towards End TB targets. Infect Dis Poverty. 2017 Jun 1; 6(1): 70. DOI: 10.1186/s40249-017-0284-4 28569191 PMC5452345

[13] Di Gennaro F, Vittozzi P, Gualano G, et al. Active Pulmonary Tuberculosis in Elderly Patients: A 2016–2019 Retrospective Analysis from an Italian Referral Hospital. Antibiotics (Basel). 2020 Aug 7; 9(8): 489. DOI: 10.3390/antibiotics9080489 PMC7459440 32784552

[14] Kristensen KL, Ravn P, Petersen JH, et al. Long-term risk of tuberculosis among migrants according to migrant status: a cohort study. Int J Epidemiol. 2020 Jun 1; 49(3): 776–785. DOI: 10.1093/ije/dyaa063 32380550

[15] Yen YF, Feng JY, Pan SW, Chuang PH, Su VY, Su WJ. Determinants of mortality in elderly patients with tuberculosis: a population-based follow-up study. Epidemiol Infect. 2017 May; 145(7): 1374–1381. DOI: 10.1017/S0950268817000152 28190404 PMC9203318

[16] Piergallini TJ, Turner J. Tuberculosis in the elderly: Why inflammation matters. Exp Gerontol. 2018 May; 105: 32–39. DOI: 10.1016/j.exger.2017.12.021 29287772 PMC5967410

[17] Ingrosso L, Vescio F, Giuliani M, et al. Risk factors for tuberculosis in foreign-born people (FBP) in Italy: a systematic review and meta-analysis. PLoS One. 2014 Apr 14; 9(4): e94728. PMID: 24733156; PMCID: PMC3986251. DOI: 10.1371/journal.pone.0094728 24733156 PMC3986251

[18] Chemtob D, Ogum E. Tuberculosis treatment outcomes of non-citizen migrants: Israel compared to other high-income countries. Isr J Health Policy Res. 2020 Aug 3; 9(1): 29. PMID: 32741367; PMCID: PMC7397670. DOI: 10.1186/s13584-020-00386-1 32741367 PMC7397670

[19] Di Gennaro F, Pizzol D, Cebola B, et al. Social determinants of therapy failure and multi drug resistance among people with tuberculosis: A review. Tuberculosis (Edinb). 2017 Mar; 103: 44–51. DOI: 10.1016/j.tube.2017.01.002 28237033

[20] Hargreaves S, Lönnroth K, Nellums LB, et al. Multidrug-resistant tuberculosis and migration to Europe. Clin Microbiol Infect. 2017 Mar; 23(3): 141–146. DOI: 10.1016/j.cmi.2016.09.009 27665703

[21] Odone A, Riccò M, Morandi M, Borrini BM, Pasquarella C, Signorelli C. Epidemiology of tuberculosis in a low-incidence Italian region with high immigration rates: differences between not Italy-born and Italy-born TB cases. BMC Public Health. 2011 May 23; 11: 376. DOI: 10.1186/1471-2458-11-376 21605460 PMC3121636

[22] Di Gennaro F, Lattanzio R, Falanga C, et al. Low-Wage Agricultural Migrant Workers in Apulian Ghettos, Italy: General Health Conditions Assessment and HIV Screening. Trop Med Infect Dis. 2021 Oct 15; 6(4): 184. DOI: 10.3390/tropicalmed6040184 34698299 PMC8544678

[23] Merid MW, Gezie LD, Kassa GM, Muluneh AG, Akalu TY, Yenit MK. Incidence and predictors of major adverse drug events among drug-resistant tuberculosis patients on second-line anti-tuberculosis treatment in Amhara regional state public hospitals; Ethiopia: A retrospective cohort study. BMC Infect. Dis. 2019; 19: 286. DOI: 10.1186/s12879-019-3919-1 30917788 PMC6437856

[24] Lan Z, Ahmad N, Baghaei P, et al. Drug-associated adverse events in the treatment of multidrug-resistant tuberculosis: an individual patient data meta-analysis. Lancet Respir Med. 2020 Apr; 8(4): 383–394. DOI: 10.1016/S2213-2600(20)30047-3 32192585 PMC7384398

[25] Noor S, Ismail M, Khan F. Drug safety in hospitalized patients with tuberculosis: Drug interactions and adverse drug effects. Clin Respir J. 2021 Jan; 15(1): 97–108. DOI: 10.1111/crj.13276 32949069

[26] Borisov S, Danila E, Maryandyshev A, et al. Surveillance of adverse events in the treatment of drug-resistant tuberculosis: first global report. Eur Respir J. 2019 Dec 19; 54(6): 1901522. DOI: 10.1183/13993003.01522-2019 31601711

[27] Holden IK, Andersen PH, Wejse C, Lillebaek T, Johansen IS. Review of tuberculosis treatment outcome reporting system in Denmark, a retrospective study cohort study from 2009 through 2014. BMC Health Serv Res. 2020 Feb 3; 20(1): 83. PMID: 32013962; PMCID: PMC6998178. DOI: 10.1186/s12913-020-4927-y 32013962 PMC6998178

[28] Peri AM, Bernasconi DP, Galizzi N, et al. Determinants of patient and health care services delays for tuberculosis diagnosis in Italy: a cross-sectional observational study. BMC Infect Dis. 2018 Dec 20; 18(1): 690. DOI: 10.1186/s12879-018-3609-4 30572830 PMC6302482

[29] Pezzotti P, Pozzato S, Ferroni E, et al. Delay in diagnosis of pulmonary tuberculosis: A survey in the Lazio region, Italy Epidemiology Biostatistics and Public Health. 2015; 1–10: 12.

[30] Koo HK, Min J, Kim HW, et al. Prediction of treatment failure and compliance in patients with tuberculosis. BMC Infect Dis. 2020 Aug 24; 20(1): 622. DOI: 10.1186/s12879-020-05350-7 32831044 PMC7446045

[31] Riccardi N, Pontarelli A, Alagna R, et al. Epidemiology and treatment outcome of MDR and pre-XDR TB in international migrants at two reference centers in the North of Italy: a cross-sectional study coordinated by Stop TB Italia Onlus. Public Health. 2020 Mar; 180: 17–21. DOI: 10.1016/j.puhe.2019.10.022 31837610

[32] Aslam MV, Owusu-Edusei K, Marks SM, et al. Number and cost of hospitalizations with principal and secondary diagnoses of tuberculosis, United States. Int J Tuberc Lung Dis. 2018; 22(12): 1495–1504. DOI: 10.5588/ijtld.18.0260 30606323 PMC8050949

[33] Nishikiori N, Van Weezenbeek C. Target prioritization and strategy selection for active case-finding of pulmonary tuberculosis: a tool to support country-level project planning. BMC Public Health. 2013; 13: 97. DOI: 10.1186/1471-2458-13-97 23374118 PMC3602078

[34] World Health Organization (WHO). Implementing the end TB Strategy. Geneva 2015. https://www.who.int/publications/i/item/implementing-the-end-tb-strategy.

[35] Saunders MJ, Evans CA. COVID-19, tuberculosis and poverty: preventing a perfect storm. Eur Respir J. 2020 Jul 9; 56(1): 2001348. DOI: 10.1183/13993003.01348-2020 32444399 PMC7243392

[36] Di Gennaro F, Marotta C, Antunes M, Pizzol D. Diabetes in active tuberculosis in low-income countries: to test or to take care? Lancet Glob Health. 2019 Jun; 7(6): e707. DOI: 10.1016/S2214-109X(19)30173-1 31097272

